# Seasonality of benign paroxysmal positional vertigo: A retrospective study from Central Europe

**DOI:** 10.1007/s00508-023-02237-w

**Published:** 2023-07-05

**Authors:** Heidemarie Zach, David Retter, Michaela Schmoeger, Paulus Rommer, Ulrike Willinger, Felix K. Schwarz, Gerald Wiest

**Affiliations:** Neurotology Outpatient Clinic, Dept. of Neurology, Medical University of Vienna, Waehringerguertel 18–20, 1090 Vienna, Austria

**Keywords:** Seasonality, Benign paroxysmal positional vertigo, Central Europe, Vertigo, Daylight, Austria

## Abstract

**Background:**

Although benign paroxysmal positional vertigo (BPPV) is the most common cause of vertigo in clinical practice, factors influencing the pathophysiology remain not fully understood.

**Objective:**

Here we aim to investigate possible seasonal influences on the occurrence of BPPV in Vienna, a city located in a Central European country with pronounced seasonal fluctuations.

**Methods:**

We retrospectively investigated data from 503 patients presenting with BPPV to the outpatient clinics of the Medical University of Vienna between 2007 and 2012. Analyses included age, gender, type of BPPV, seasonal assignment, as well as daylight hours and the temperature in Vienna at symptom onset.

**Results:**

Out of 503 patients (159 male, 344 female, ratio 1:2.2; mean age 60 ± 15.80 years), most patients presented with posterior (89.7%) and left-sided (43.1%) BPPV. There was a significant seasonal difference (χ^2^ *p* = 0.036) with the majority of symptoms occurring in winter seasons (*n* = 142), followed by springtime (*n* = 139). Symptom onset did not correlate with the average temperature (*p* = 0.24) but on the other hand very well with daylight hours (*p* < 0.05), which ranged from 8.4 h per day in December, to an average of 15.6 h in July.

**Conclusion:**

Our results show a seasonal accumulation of BPPV during winter and springtime, which is in line with previous studies from other climatic zones, suggesting an association of this seasonality with varying vitamin D levels.

## Introduction

Benign paroxysmal positional vertigo (BPPV) is the most common cause of vertigo seen in balance clinics, with a lifetime prevalence of up to 2.4% [1]. Although BPPV can occur at any age, the peak age is around 60–70 years. Women are generally more often affected than men, with a gender ratio (men:women) of around 1 : 1.5–2 [2]. BPPV is characterized by episodes of often highly debilitating vertigo spells with nystagmus and nausea, triggered by changes of head or body positions [2, 3].

Although the pathophysiology still remains not fully understood, the widely accepted cause of BPPV is explained by displaced otoconia falling from the utricular macula into a semicircular canal. Triggered by head movements, freely floating otoconia (canalolithiasis) or adherent deposits on the cupula (cupulolithiasis) react to head rotation or gravity, inducing shearing forces on the cupula [4]. Special maneuvers, such as the Epley and the Semont maneuver for posterior BPPV and the barbecue roll or Gufoni maneuver for affection of the horizontal canals are state of the art treatment options for the repositioning of dislodged otoconia and show a success rate of up to 90% [5–8]. The majority of BPPVs are idiopathic, while about 5% are symptomatic, with trivial head trauma as the most common cause [9], followed by inflammation, including viral labyrinthitis and vestibular neuritis. Furthermore, associations with migraine, Ménière’s disease, hypertension and bed confinement have been proposed [10, 11].

The higher incidence of BPPV in older people has been linked to the progressive degeneration of the macula with increasing age. Additionally, there are associations with osteoporosis, female gender and vitamin D deficiency, as otoconia are primarily composed of calcium carbonate crystals, arranged around an organic core [12]. Similar to the metabolism of bone substance, vitamin D plays an important role in the development of otoconial structure, explaining the possible association of vitamin D deficiency and occurrence of BPPV [12–15]. Recently a large randomized trial from South Korea revealed a prophylactic effect of vitamin D supplementation [16]. Vitamin D deficiency is most prominent in wintertime and, although controversially discussed, some studies have argued the peak of neurotrophic viral infections to be also in winter and spring [17, 18] as well as associations with barometric pressure, migraine and poor physical activity [19–23]. Hence, a correlation between seasonality and significantly lower BPPV incidence with temperature increase has been described before [21, 24–27].

A predominance of cases with right-sided BPPV, as opposed to the left side, has been proposed but this could potentially be explained by preferred sleeping positions [28, 29]. Given its anatomic characteristics, the posterior canal is the most affected as its inferior position facilitates otoconia to drop into the canal during side sleeping and moving to the bottom when sitting up; however, the estimated incidence of horizontal BPPV might be higher than reflected in the number of patients visiting outpatient clinics. Given the orientation of the horizontal canal, spontaneous repositioning of the particles seems plausible, for instance when patients involuntarily imitate the barbecue reposition maneuver by turning in bed during sleep [30, 31].

In this retrospective observational study, we investigated cases of patients presenting with BPPV to our outpatient clinics within a period of 6 years. We evaluated epidemiological factors including age, gender, characteristics of BPPV, as well as seasonality (including assessment of temperature and daylight hours at onset). To our knowledge, this is the largest study on the seasonality of BPPV in Central Europe, which, in contrast to Mediterranean countries, is classified as having a continental climate, featuring hot summers, cold winters, and large annual ranges of temperatures, which predestines Vienna as an ideal climate zone to study the seasonality of diseases.

## Material and methods

### Study population and seasonality

We retrospectively evaluated the charts of all patients presenting with vertigo to the Neurotology Outpatient Clinic, the Neurology Outpatient Clinic and the Department of Emergency Medicine at the Medical University of Vienna, between January 2007 and December 2012. Only patients > 18 years with definite BPPV (based on the ICD-10 criteria H81.1) and diligently documented symptoms, were included. At the emergency department and in general at the AKH-Vienna (University Hospital Vienna), patients are usually investigated in an interdisciplinary setting. Therefore, almost 90% of vertigo patients are either seen first by the ENT or experienced neurologists and are then referred to colleagues from the other specialty who are familiar with the diagnosis and treatment of BPPV. In addition, all cases were supervised by specialists from the Neurotology Outpatient Clinic. Treatment-resistant cases were also reassigned to the Neurotology Outpatient Clinic for diagnosis confirmation and further treatment. As the patients were admitted in the acute stage, the maneuvers were performed either by consultants or residents experienced in positioning (at the end of the training under supervision). Due to the random distribution of patients (admissions during the daytime, or at night, or at the weekend, i.e., 24 h for 7 days a week), the maneuvers were not tied to a single investigator.

Patients with any signs of other causes of peripheral or central vertigo were not included in this study. Based on the meteorological seasons in Austria, spring begins in March, summer in June, autumn in September and winter in December. The average temperatures, as well as the daylight hours in the particular months, were provided by the Central Institute for Meteorology and Geodynamics of Austria (ZAMG).

### Statistical analysis and calculation

Descriptive statistics were used to describe patient characteristics as well as the number of outpatient visits at the Medical University of Vienna across the seasons, χ^2^-tests were applied to assess differences in the occurrence of BPPV and Spearman’s correlation coefficients were used to evaluate significant correlations between the occurrence of BPPV and daylight hours. To investigate the influence of air temperatures during 2007–2012, we evaluated the differences of BPPV frequencies between extremely cold and extremely warm months (mean temperature ±1 standard deviation, SD), using χ^2^-tests.

The study was approved by the local Ethics Committee of the Medical University of Vienna and was performed according to the standards of the 1964 Declaration of Helsinki. Statistical analyses were performed using the IBM SPSS 25.0 (SPSS, IBM SPSS Inc., Chicago, IL, USA) software.

## Results

### Patient characteristics

**Fig. 1:**
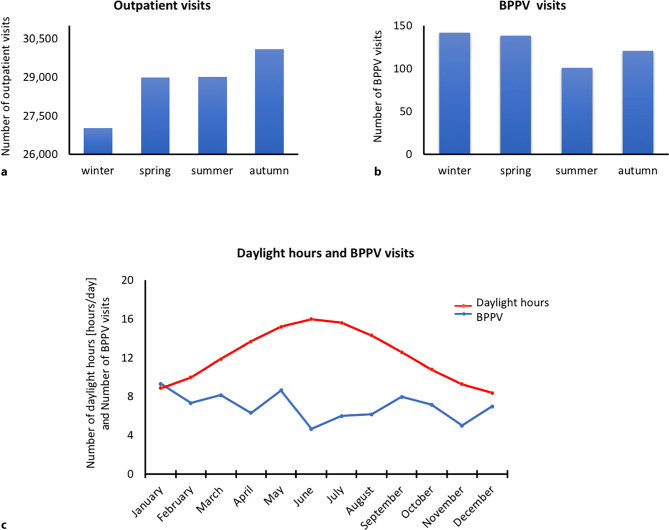
Seasonal influences on the occurrence of benign paroxysmal positional vertigo (BPPV). Diagrams showing the seasonal distribution of patient visits during all 6 years (January 2007–December 2012) in spring (March, April, May), summer (June, July, August), autumn (September, October, November) and winter (December, January, February). The total numbers of patients presenting to the outpatient clinics of Neurotology Outpatient Clinic, the Neurology Outpatient Clinic and the Department of Emergency Medicine at the Medical University of Vienna are shown in (**a**). The seasonal distribution of BPPV visits (*n* = 503) is shown in (**b**). **c** Graph showing the average daylight hours per day during the course of 1 year (Y-axis: daylight hours/day; X‑axis: months) represented by the *red line*. The average number of BPPV visits cases per month (Y-axis: number of BPPV visits; X‑axis: months) are represented by the *blue line*. All results in (**c**) are averaged across the study period of 6 years (2007–2012)

Between January 2007 and December 2012 in total 115,146 patients were seen by neurologists during their presentation at the Neurology Outpatient Clinic, the Neurotology Outpatient Clinic and the Department of Emergency Medicine at the Medical University of Vienna (see Fig. 1a). Of this group of patients with all kinds of neurological symptoms, 503 patients (mean age 60 ±15.8 years, male *n* = 159, male to female ratio 1:2.2) presented with definite BPPV.

### Seasonality of BPPV occurrence

Within the study period of 6 years a significant difference in the seasonal frequency of BPPV was observed (χ^2^(3) = 8.55; *p* = 0.038). Most cases occurred during wintertime (*n* = 142; 28.2%), followed by springtime (*n* = 139; 27.6%) whilst the least cases of BPPV were reported during summer (*n* = 101; 20.1%) and autumn (*n* = 121; 24.1%; see Table 1 and Fig. 1b).

**Table 1:** Patient frequencies and BPPV occurrences across the seasons (January 2007–December 2012)

	BPPV occurrence		Outpatient visits	
Winter (*n*;%)	142	28.2%	27,035	23.5%
Spring (*n*;%)	139	27.6%	29,007	25.2%
Summer (*n*;%)	101	20.1%	29,017	25.2%
Autumn (*n*;%)	121	24.1%	30,087	26.1%
**Total (*n*)**	**503**	**–**	**115,146**	–

*BPPV occurrence* total number of patients presenting with BPPV; *Outpatient visits* total number of patients presenting to the Neurotology Outpatient Clinic, the Neurology Outpatient Clinic and the Department of Emergency Medicine at the Medical University of Vienna

### Seasonality of outpatient visits

Within the relevant time period the most visits of patients seen by neurologists at our outpatient clinics occurred in autumn (*n* = 30,087, 26.1%) followed closely by spring (29,007, 25.2%) and summer (29,017; 25.2%) (with almost equal amounts) and ultimately by winter, which garnered the least visits (27,035; 23.5%, Fig. 1a).

### Average daylight hours in Vienna at symptom onset

In the years 2007–2012 average daylight hours in Vienna ranged from 8.4 h per day in December, to an average of 15.6 h in July (see Fig. 1c). The correlation of the mean daylight hours/day of the particular months with BPPV occurrence showed a negative trend (ρ = −0.17, *p* = 0.078), when correlating with daylight hours of the preceding month (e.g., daylight hours in January correlated with BPPV cases in February) the negative correlation reached significance (ρ = −0.21, *p* = 0.042) with significantly more BPPV cases during darker months.

### Average temperature in Vienna at symptom onset

To investigate potential influence of air temperature in Vienna, the frequency of BPPV was correlated with the average temperature in the particular months. The mean temperature in 2007–2012 was 11.1 °C (SD = 7.6°). During extremely cold months (temperature < 3.5 °C) more BPPV cases (*n* = 113) occurred than during extremely warm months (temperature > 18.8 °C; *n* = 96), although without reaching significance (χ^2^(1) = 1.4; *p* = 0.24).

### BPPV characteristics

**Fig. 2:**
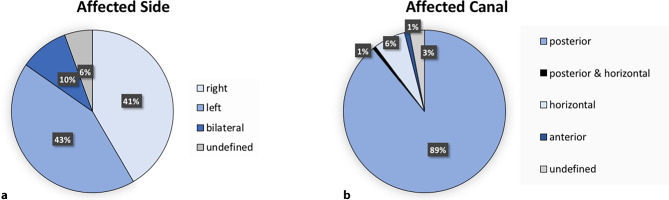
Characteristics of benign paroxysmal positional vertigo presentations. **a** Pie chart showing the distribution of the affected canal side in percentage (*n* = 503), with a majority of left-sided BPPVs (43%; *blue*), followed by the right side (41%, *light blue*), bilateral affection (10%; *dark blue*) and undefined side (6%; *grey*). **b** Pie chart showing the distribution of the affected canals, with a majority of the posterior BPPV variant (89%; *blue*), followed by the horizontal canal (6%; *light blue*), the anterior canal (1%; *dark blue*), concurrent affection of the posterior and horizontal canals (1%; *black*) and undefined canal (3%, *grey*). “Undefined” side or canals refers to rare cases where the definite diagnosis of BPPV has been established by the examiner, but the affected side or canal has not been further documented in the clinical report

Of the 503 BPPV patients, 217 presented with left-sided BPPV, while in 209 patients the right side was affected, 49 patients had bilateral BPPV, whilst the side was not defined in the remaining 28 patients (see Fig. 2a). The majority of the 503 BPPV patients presented with posterior canal BPPV (*n* = 448). In 32 patients, the horizontal canal was affected, whereas 3 patients presented with both posterior and horizontal BPPV. The anterior canal was affected only in 5 patients and in 15 patient charts the canal was not defined (see Fig. 2b).

## Discussion

In this study we retrospectively investigated the seasonal incidence, epidemiological factors and clinical characteristics in patients presenting with BPPV to the involved outpatient clinics of the Medical University of Vienna. The main findings of our study were (i) a significant correlation of BPPV with seasons and daylight hours (ii) a female preponderance in BPPV patients and (iii) a preponderant affection of the left posterior canal.

### Seasonality and daylight

Within the study period of 6 years, the number of BPPV-associated visits to our outpatient clinics in Vienna was significantly higher during winter and springtime and lowest during summertime, with a negative correlation between daylight hours and the occurrence of BPPV. The correlation of daylight hours during the preceding months with the occurrence of BPPV reached significance. In light of the previously suspected link between BPPV, seasonal influences and subsequent fluctuations in vitamin D levels, we shifted the timeframe of the correlation by 1 month as vitamin D synthesis will result in altered vitamin D levels, with corresponding impact in the following months. During wintertime, daylight hours in Vienna are limited to about 8.5 h, thus leading to reduced vitamin D synthesis in the skin. In summertime, the mean daylight hours are almost twice (about 15.5 h) as long as in winter, which increases the opportunity to replenish the vitamin D storage. This explains the seasonal fluctuations of vitamin D levels and serum calcium concentrations in Austria, which show a pattern similar to daylight hours, with lowest levels in February and a peak in August [17, 32, 33]. The findings of our study, being so far the largest study from Austria in Central Europe to our knowledge, are in line with data from previous studies conducted all over the world, which found the same pattern of seasonality in BPPV [1, 13, 24–26, 32, 34].

BPPV episodes occurred more often in the coldest months, but the correlation between mean temperature in Vienna and the months of BPPV onset did not reach significant levels. A study in Italy showed a significant negative correlation with temperature and the occurrence of BPPV. The authors reported that a temperature increase of 1 °C reduced BPPV visits by around one third [24]; however, due to the location in different climate zones of Austria and Italy, the data cannot be directly compared. In addition, a recent study on the increased incidence of BPPV during the COVID lockdown in Italy established a link between BPPV and low levels of physical activity, which could be the case in a city like Vienna during the winter season [22].

### Epidemiology

In our cohort, more than twice as many women than men had BPPV (159 men, 344 women), with a men:women ratio of 1:2.2, which goes in line with prior research [1, 2]. Hormonal influences, especially estrogen in combination with vitamin D, play an important role in the calcium and phosphorus metabolism of bone mineral density and the calcium carbonate crystals of the otolithic membrane [3, 34, 35]. Remodeling processes in the inner ear can be worsened by estrogen deficiency, which has been speculated to increase the risk of BPPV. This hypothesis is also supported by the study from Vibert et al., which has shown ultrastructural changes of otoconia along with decreased bone mineral density in osteoporotic rats after ovariectomy [36].

The mean age of patients presenting with BPPV in our study was 60 years, with the oldest patient being a 103-year-old man and the youngest being a 20-year-old man. Clinical experience shows that BPPV can occur at any age, albeit with great variety, but the likelihood of BPPV increases with age [34, 37]. Degenerative processes have been proposed to be responsible for progressive detachment of the otoconia during aging, leading to BPPV at a certain point [38].

### Characteristics of BPPV

Out of the 503 BPPV patients, the left side was slightly more (43.1%) affected than the right side (41.1%), although in 5.6% the side was not defined and in 9.7% bilateral involvement was reported (see Fig. 2a). Prior studies have found a slight predominance of the right side, which has been attributed to the patients’ preferred sleeping positions [28, 29, 39].

Consistent with the literature, most of our patients presented with BPPV of the posterior semicircular canal (89.1%; see Fig. 2b). Anatomical features of this canal are the main cause for the frequent occurrence of this BPPV variant, as sleeping in a supine position facilitates detached otoconia to enter the posterior semicircular canal [29, 40]; however, the chance of spontaneous reposition of the otoconia by involuntary head movements during daily life is lower in the posterior canal than in, for example, the horizontal canals, which is in line with the lower number of the horizontal BPPV variant (6.3%) in our cohort (see Fig. 2b; [41]). Here, spontaneous remission of dislocated otoconia is possible during involuntary turning in bed, similar to the barbecue maneuver and this might not even be recognized by patients. The anterior canal was least affected, with it making up less than 1% of cases. This could also be explained by the anatomical position of the canal, as it reaches beyond the vestibulum, preventing otoconia to drop into this canal. Although there is some agreement of a lower rate of involvement of the anterior canal, the reported incidence rates range from 1% to even 21% and therefore require further studies [42]. In 15 patients of our study, the affected semicircular canals were not defined. In these cases, we retrospectively cannot exclude simultaneous affections of multiple canals with changing patterns of nystagmus, which may have impeded the diagnosis [43].

### Limitations and interpretation

As patients with BPPV do not exclusively present to our Neurotology Outpatient Clinic, but also to the Neurology Outpatient Clinic and the Department of Emergency Medicine at the Medical University of Vienna we investigated all outpatient visits of those departments. Here we could not exclude that the frequency of outpatient visits during summer, the holiday season in Austria, might have skewed the number of BBPV presentations; however, contrary to the occurrence of BPPV, the number of outpatient visits was even higher in summer than during wintertime, which invalidates the argument set out above (see Fig. 1a, b).

In general, epidemiological studies are often limited as no definite causality can be drawn. In our study neither vitamin D levels nor individual daylight exposures were taken into consideration and should be evaluated in future prospective studies in Europe. Furthermore, we did not evaluate physical activity, barometric pressure or migraine within this study, as well as multimorbidity and increased level of anxiety due to the vertigo which could be additional factors for seasonality and should be part of future studies.

However, we believe that our large cohort size, so far the largest study from Central Europe to our knowledge, and the evaluated findings, which are in line with several prior studies, add to previously published evidence of the seasonality of BPPV.

## Conclusion

Our findings correlate with previous studies from countries around the world, where the potential influence of cold seasons, vitamin D deficiencies, female gender and age on the occurrence BPPV has been shown [3, 13, 14, 18, 21, 24, 25, 27, 32, 38]. In the assumption that otoconia and bones share a similar mineralization metabolism, of which vitamin D is an integral part, risk factors for reduced bone mineral density have also been linked to BPPV [16, 44–46]. The role of vitamin D regarding the mineralization of otoconia is currently increasingly discussed. Further studies are needed to evaluate possible prophylactic interventions, such as the effects of seasonal cholecalciferol or calcium supplementation in the future.
